# Controlling Rater Effects in Divergent Thinking Assessment: An Item Response Theory Approach to Individual Response and Snapshot Scoring

**DOI:** 10.3390/jintelligence13060069

**Published:** 2025-06-17

**Authors:** Gerardo Pellegrino, Janika Saretzki, Mathias Benedek

**Affiliations:** 1 Department of General Psychology, University of Padova, 35131 Padova, Italy; 2 Department of Psychology, University of Graz, 8010 Graz, Austria; janika.saretzki@uni-graz.at; 3 Department of Psychology, Charlotte Fresenius Hochschule München, 80797 Munich, Germany; 4 Department of Psychology, LMU Munich, 80802 Munich, Germany

**Keywords:** item response theory, judge response theory, creativity assessment, divergent thinking assessment, consensual assessment technique

## Abstract

Scoring divergent thinking (DT) tasks poses significant challenges as differences between raters affect the resulting scores. Item Response Theory (IRT) offers a statistical framework to handle differences in rater severity and discrimination. We applied the IRT framework by re-analysing an open access dataset including three scored DT tasks from 202 participants. After comparing different IRT models, we examined rater severity and discrimination parameters for individual response scoring and snapshot scoring using the best-fitting model—Graded Response Model. Secondly, we compared IRT-adjusted scores with non-adjusted average and max-scoring scores in terms of reliability and fluency confound effect. Additionally, we simulated missing data to assess the robustness of these approaches. Our results showed that IRT models can be applied to both individual response scoring and snapshot scoring. IRT-adjusted and unadjusted scores were highly correlated, indicating that, under conditions of high inter-rater agreement, rater variability in severity and discrimination does not substantially impact scores. Overall, our study confirms that IRT is a valuable statistical framework for modeling rater severity and discrimination for different DT scores, although further research is needed to clarify the conditions under which it offers the greatest practical benefit.

## 1. Introduction

Divergent thinking (DT) represents the ability to find new and original ideas for open-ended problems and is considered one of the main indicators of creative potential. Since Guilford’s (1950, 1967) seminal work, DT tasks have become some of the most commonly used tools in creativity research, as they offer a practical way to assess individuals’ capacity for idea generation—a central component of creative cognition (Reiter-Palmon et al. 2019). However, while DT plays a key role in creative thinking, it represents only one aspect of the broader creative process, which also involves other components such as problem identification, idea evaluation, and implementation (Botella et al. 2018; Lubart 2001). Therefore, DT scores should not be interpreted as comprehensive measures of creativity. Nonetheless, performance in DT tasks is predictive of real-life creative achievements (Benedek 2024; Said-Metwaly et al. 2022), which explains their widespread use in creativity research.

Different DT tasks have been developed, and the Alternate Uses Task (AUT) is one of the most frequently used (for a review, see Saretzki et al. 2024a). The AUT asks participants to generate creative uses for everyday objects (e.g., a brick) within a limited time. The performance in DT tasks can be scored with respect to the quantity and quality of responses, which are typically assessed in terms of DT fluency (i.e., number of responses), DT originality or creativity (i.e., creative quality of the responses), and DT flexibility (i.e., number of different categories or shifts between ideas). While fluency and flexibility scoring are more straightforward, scoring originality can be challenging, as it typically requires different raters who evaluate the answers provided by participants and assign them a score. Thus, scoring originality requires time and resources, especially in research designs involving multiple DT tasks and large sample sizes (Forthmann et al. 2017a, 2023). For this reason, much of the effort from creativity researchers has been devoted to investigating the best (and less burdensome) ways of assessing originality that can produce reliable and robust results (Forthmann et al. 2023).

In particular, research has focused on improving the inter-rater agreement between raters assessing DT tasks (Ceh et al. 2022; Forthmann et al. 2017a). According to the Consensual Assessment Technique (Amabile 1982; Baer et al. 2004), a response can be considered creative if independent raters agree that it is creative. However, this approach is highly dependent on the characteristics of the raters, who may vary in their leniency vs. strictness (i.e., over- or underestimation of originality) and discernment (i.e., the ability to discriminate between the originality of different responses; Benedek et al. 2016; Ceh et al. 2022; Silvia 2008). For this reason, recent studies have attempted to account for rater variability using advanced statistical paradigms that incorporate rater discrepancies to derive more reliable estimates of originality. One such framework is Item Response Theory (IRT; see Myszkowski and Storme 2019; Primi et al. 2019, for applications in creativity research).

Drawing from the large IRT literature, Myszkowski and Storme (2019) have proposed the Judge Response Theory (JRT), which accounts for differences between judges in rating the DT tasks. JRT is a powerful tool for analysing raters’ variability and estimating originality factor scores (i.e., an adjusted score that takes into account differences between raters) that can be used in further analysis (Myszkowski 2021). Although the JRT framework represents an important advance for creativity researchers (Barbot et al. 2023; Forthmann et al. 2023; Myszkowski and Storme 2019), further research is still needed to quantify the extent to which its application influences results compared to more traditional approaches. While IRT is theoretically more accurate, it remains valuable for us to evaluate whether adopting IRT models in a research design leads to substantially different outcomes. Doing so can help researchers make more informed decisions about whether to adopt IRT-based methods or rely on classic approaches. For example, it is possible that IRT models offer different benefits when applied to different scoring procedures, such as individual response scoring and snapshot scoring. Additionally, IRT provides valuable insights into rater characteristics, which can deepen researchers’ understanding of their data.

### 1.1. From Individual Response Scoring to Snapshot Scoring

Different methods of originality scoring have been developed over the years (Reiter-Palmon et al. 2019; Saretzki et al. 2024b). Scoring methods involving human raters can be divided into two main approaches: individual response scoring and snapshot scoring (Reiter-Palmon et al. 2019; Silvia 2011; Silvia et al. 2009). In individual response scoring, each rater provides scores for each creative idea in a task, which are then aggregated for further analysis. In the snapshot scoring, raters are asked to evaluate each participant’s entire pool of answers and assign them an overall score. Recent originality scoring procedures do not involve human raters (for some examples, see Patterson et al. 2023; Yu et al. 2023; Zielińska et al. 2023). However, these scoring methodologies are not the focus of the present research.

In individual response scoring, different aggregation procedures are possible once the raters have evaluated all the responses. Summative scoring consists of simply summing all the scores obtained by participants’ responses. However, this procedure has been criticized for having a strong confounding effect with fluency (Clark and Mirels 1970; Forthmann et al. 2021): Participants may appear more original simply by giving more answers, even if their originality is low. A second and widely used aggregation method is the average scoring (or ratio scoring; Plucker et al. 2011), which represents the average of all individual response scores. While this approach significantly reduces the fluency confounding effect, it is still affected by variability in task strategies (Benedek et al. 2013). Indeed, some individuals may generate and report every idea that comes to their mind, even the less-creative ones, while other individuals may focus on reporting only the most creative ideas. However, with average scoring, the first may be penalized compared to the latter as including non-creative ideas lowers their average score (Saretzki et al. 2024b). One way to overcome this limitation is the use of max-scoring (Smeekens and Kane 2016). For example, a max-3 score reflects the average evaluation of the three responses with the highest ratings. In this way, participants who reported many responses are not penalized by their less-creative responses. However, it is up to the researcher to establish the maximum number of responses that should be considered, and this decision can significantly impact the results (Saretzki et al. 2024b).

Unlike individual response scoring, snapshot scoring involves raters scoring the entire ideational pool—all responses a person gave in a task—at once (Silvia et al. 2009). Thus, a single holistic rating is attributed to the ideational pool, which reflects an implicit response aggregation and thus represents yet another way to avoid the fluency confound. While snapshot scoring may seem less effortful and time-consuming than individual response scoring, it can still be a demanding task (Forthmann et al. 2017a). The pools of ideas for each participant can be wide and elaborate and may encompass ideas with different degrees of originality, making it difficult to provide a single global score. Moreover, raters may differ in how they weigh the number of ideas or the diversity of responses in their total scores (Forthmann et al. 2017a).

Although different, individual response scoring and snapshot scoring share a common characteristic: They assume that raters are assessing the tasks in the same (or, at least, comparable) way. In other words, raters are expected to give similar ratings to the same responses or ideational pools, and a high level of inter-rater agreement or consistency (as calculated, for example, by the Intraclass Correlation Coefficient) is considered fundamental to the reliability and interpretation of the scores. For this reason, rater training is an essential part of the scoring of DT tasks. During this phase, raters are instructed about the criteria to follow while attributing the scores. While providing accurate instructions is fundamental to increasing inter-rater reliability in DT task scoring (Forthmann et al. 2017a; Silvia et al. 2008), individual differences across raters may still have an impact on their evaluations (Ceh et al. 2022).

Given that inter-rater variability can only be controlled to a limited extent, recent studies have attempted to focus on how to actively account for inter-rater variability from a psychometric perspective and how to obtain more reliable measures of originality. JRT (Myszkowski and Storme 2019) may provide a psychometric framework to better control for rater differences, thereby making DT scoring procedures more reliable and effective.

### 1.2. Item Response Theory Applied to DT Scoring

JRT represents a recent application of IRT to DT task scoring. This framework was put forward by Myszkowski and Storme (2019), and the open access R package jrt was developed to make these analyses more accessible to researchers (Myszkowski 2021). JRT draws from well-established IRT models used for ordinal and polytomous variables (such as the Likert scales which are traditionally used in DT scoring), including the Graded Response Model (Samejima 1968), the Rating Scale Model (Andrich 1978), the Partial Credit Model (Masters 1982), and the Generalized Partial Credit Model (Muraki 1993). These models are largely used with ordinal scales as they compute the probability of selecting a certain response category or higher on an item (i.e., step function). In the case of DT tasks, JRT analyses can be used to model the likelihood of a rater giving a certain score or higher. The jrt package (Myszkowski 2021) allows for estimating and comparing all these models at once, to easily identify which one fits the data best through information criteria.

JRT models are useful for dealing with the possibility that raters vary in their severity, in their ability to discriminate between ideas, and in their tendency to use the ordinal response scale in different ways (e.g., tendencies to either use central or extreme ratings). Raters’ severity and discrimination are included as parameters used to calculate latent factors scores (Myszkowski 2021; Myszkowski and Storme 2019). However, this recent framework has been used in only a few studies (Barbot et al. 2023; Forthmann et al. 2023), and the extent to which JRT-adjusted scores may differ from the non-adjusted ones is still unknown. In other words, it is not clear under which conditions applying JRT enhances the reliability of DT tasks scores. Additionally, existing applications of JRT have focused exclusively on ratings of individual responses, despite its potential applicability to ideational pools, such as those used in snapshot scoring.

It should be noted that JRT models only allow differences between raters to be examined. However, DT data are often more complex, as researchers usually employ different tasks in their experimental settings. For example, when administering the AUT, participants are usually presented with different objects. In a previous attempt to control for raters’ variability, Primi et al. (2019) applied the Many-Facet Rasch Model (MFRM) for polytomous ratings (Eckes 2011) on tasks of metaphors, alternate uses, and creative instances productions. The MFRM allows not only to quantify and adjust different raters’ severity, but also to account for scores in multiple tasks (for example, in this study, the AUT included three objects: can, knife, and hairdryer). While in this study the MFRM proved to be a powerful means of modelling rater differences in DT tasks, this model has not been formally compared with the models included in the JRT framework.

Another important practical implication of this psychometric framework is that it allows for controlling missing data (Forthmann et al. 2023; Primi et al. 2019). Whereas in classical research designs raters are instructed to score all individual responses or pools of ideas, thanks to JRT models (Forthmann et al. 2023) and MFRM (Primi et al. 2019), it is possible to plan in advance for the presence of missing data (i.e., each rater will evaluate only a portion of the individual responses or ideational pools). Planned missing data designs can be extremely beneficial in terms of time and costs for creativity researchers (Forthmann et al. 2023).

### 1.3. Rationale of the Study

From a theoretical perspective, applying IRT models to DT data seems promising for overcoming several challenges that creativity researchers face, such as controlling for individual differences among raters (Ceh et al. 2022) and addressing the time and cost required for rating open-ended questions (Forthmann et al. 2023; Primi et al. 2019). IRT offers a statistical framework that can help researchers better manage DT data by accounting for potential rater differences and producing more reliable estimates of DT scores (Myszkowski and Storme 2019). Moreover, this approach can support researchers in planning for missing data, potentially reducing time, costs, and raters’ cognitive burden (Forthmann et al. 2023; Primi et al. 2019).

In the present study, we aimed to compare different DT scoring approaches using an open access dataset that included rated AUT data with individual and snapshot scores (Forthmann et al. 2017b). While the IRT framework is a theoretically robust approach to modeling DT scoring, it is also useful to empirically examine how its application compares to more traditional scoring methods, to quantify the extent to which different scoring approaches yield divergent results. This allows researchers to better understand how methodological choices may impact findings in practice. The present study has three main aims.

Aim 1. We aimed to identify the best-fitting IRT model for both individual response and snapshot scoring. Although individual response scoring and snapshot scoring share a similar structure (i.e., ordinal responses), they place different demands on raters. In individual response scoring, raters evaluate each idea separately, whereas in snapshot scoring, they assign a single score to the entire ideational pool. These differences may lead raters to vary in severity and discrimination across scoring methods, and IRT models can help quantify such similarities and differences. Moreover, it is possible that different IRT models may provide the best fit depending on the scoring procedure used.

Different IRT models can be used to model polytomous items (Myszkowski and Storme 2019), and they can be easily compared using the jrt package (Myszkowski 2021). However, the jrt package does not implement IRT-based models that account for and quantify variability both between raters and between tasks. For this reason, we compared JRT models with the MFRM previously applied by Primi et al. (2019). Once we identified the best-fitting model for both individual response scoring and snapshot scoring, we examined raters’ severity and discrimination for both scoring procedures, to underline potential similarities or peculiarities in raters’ behaviors across the two different scoring procedures.

Aim 2. We assessed the reliability of the latent DT originality factors derived from several popular scoring approaches: IRT-adjusted and unadjusted versions of average scoring (for individual response scoring and snapshot scoring), and max-scoring (focused on the best 1 to 5 responses). In this way, we aimed to highlight potential differences in reliability across different scoring approaches and evaluate how reliability differs between IRT-adjusted and non-adjusted scores. Additionally, we examined the extent of fluency confound effect for the different scores.

Aim 3. Finally, we tested the robustness of IRT-adjusted scores by simulating a scenario with missing data to identify which approach maintains consistent results in the presence of missing data (Primi et al. 2019). With this approach, we also aimed to enhance comparability between individual response scoring and snapshot scoring by reducing the number of raters in both conditions to two. We expected IRT-adjusted scores in the simulated dataset to maintain a high correlation with the original scores in the complete dataset, indicating that IRT allows the estimation of reliable factor scores even under missing data conditions. This is particularly relevant for researchers, as adopting a missing data design can reduce both the time and cost associated with scoring procedures (Forthmann et al. 2023).

## 2. Materials and Methods

### 2.1. Participants

The present study involves a reanalysis of a previously collected dataset, following a Special Issue call of this journal. The original dataset included 202 participants. One participant was excluded from our analysis because they were missing data in two out of the three DT tasks. Our final sample included 201 participants (58 males; M_age_ = 24.51; SD_age_ = 6.81). Further information on the sample can be found in Forthmann et al. (2020), Forthmann et al. (2017b), and Forthmann and Doebler (2022).

### 2.2. Creativity Measures

Participants completed three AUTs, with 2.5 min allotted per task: garbage bag, paperclip, and rope. Hybrid instructions to be both fluent and creative were given: “Please try to write down as many uncommon and creative uses for a [object-prompt] as you can think of.”. The participants overall provided 4752 valid responses across the tasks (1648 for the garbage bag, 1429 for the paperclip, and 1675 for the rope). Concerning the individual response scoring, three raters evaluated each response on a scale ranging from 1 (not at all creative) to 5 (highly creative). Three criteria were followed when evaluating the tasks: uncommonness, remoteness, and cleverness (Silvia et al. 2008). Additionally, the tasks were scored by five raters following a snapshot procedure (Silvia et al. 2009). For the ideational pools, the same 5-point Likert scale was used.

Inter-rater reliability, assessed through the Intraclass Correlation Coefficient ICC(3,k), was adequate (Koo and Li 2016) for both individual response scoring (garbage bag: ICC = 0.86, 95% CI: [0.82; 0.89]; paperclip: ICC = 0.84, 95% CI: [0.80; 0.87]; rope: ICC = 0.91, 95% CI: [0.88; 0.93]) and snapshot scoring (garbage bag: ICC = 0.91, 95% CI: [0.89; 0.93]; paperclip: ICC = 0.90, 95% CI: [0.87; 0.92]; rope: ICC = 0.88, 95% CI: [0.86; 0.91]). Further details on the tasks and the scoring procedure can be found in the original studies (Forthmann et al. 2017b; Forthmann and Doebler 2022).

Based on the individual response scoring, we computed average scores by averaging across the rating of all responses per task. Moreover, we calculated max scores by considering the 1 to 5 most creative responses per task (Saretzki et al. 2024b). For a max-n score, we selected the n individual responses of a task that had received the highest ratings and averaged across these responses. If participants reported less than n responses, the average score was calculated by considering only the responses provided (e.g., if a participant provided only 3 responses, their max-3, max-4, and max-5 scores would be the same). Finally, average scores, max scores, and snapshot scores were averaged across the three DT tasks.

### 2.3. Analysis Strategy

All analyses were performed using R (version 4.3.3; R Core Team 2024). The R script for all analyses and the Supplementary Materials are available at https://osf.io/abpnr/ (created on 21 March 2025). Data are available at https://osf.io/a9qnc/ (accessed on 27 September 2024).

Aim 1. Our first aim was to determine the best IRT model for individual response scoring and snapshot scoring. We began by checking the assumption of unidimensionality (Zielińska et al. 2022) using parallel analysis and by calculating the ratio of the first to second eigenvalue (with a ratio greater than 4:1 indicating unidimensional data).

Different commonly used IRT models for ordinal data were compared using the jrt() function (for a complete list, see Myszkowski 2021). We also ran a MFRM (Primi et al. 2019) using the TAM package (Robitzsch et al. 2024). All models included in jrt() and MFRM were compared via the Akaike Information Criterion (AIC) and the Bayesian Information Criterion (BIC), where lower AIC and BIC values indicate a better fit (Barbot et al. 2023; Forthmann et al. 2023). We compared the models separately for individual response scoring and snapshot scoring to identify the best-fitting model for each procedure.

We examined judges’ infit and outfit parameters, which indicate how well their scores align with the expectations of the IRT model. We considered values between 0.6 and 1.4 as acceptable (Wright and Linacre 1994). To compare raters’ severity (i.e., the difficulty parameter) and discrimination across individual response scoring and snapshot scoring, we plotted Judge Category Curves (JCC) and inspected their parameters. We looked at whether these curves were sorted or unsorted in both approaches. Finally, we examined the Test Information Function (TIF) for each model, which indicates the levels of the latent originality trait at which raters provide the most information. Greater information indicates a more precise measurement of the latent trait (θ), meaning that raters can make finer distinctions among responses or ideational pools in that range. Finally, we computed factor scores using Expected A Posteriori (EAP) estimates, which were used in subsequent analyses.

Aim 2. for our second aim, we examined the psychometric properties of different DT scoring methods, both with and without IRT adjustments. Before estimating each model, we standardized the observed variables (note that the factor scores extracted through IRT were already standardized). We first assessed internal consistency using Cronbach’s α, McDonald’s ω, and Hancock’s H (Saretzki et al. 2024b). EAP estimates were used to assess internal consistency of IRT-adjusted scores.

**Figure 1:**
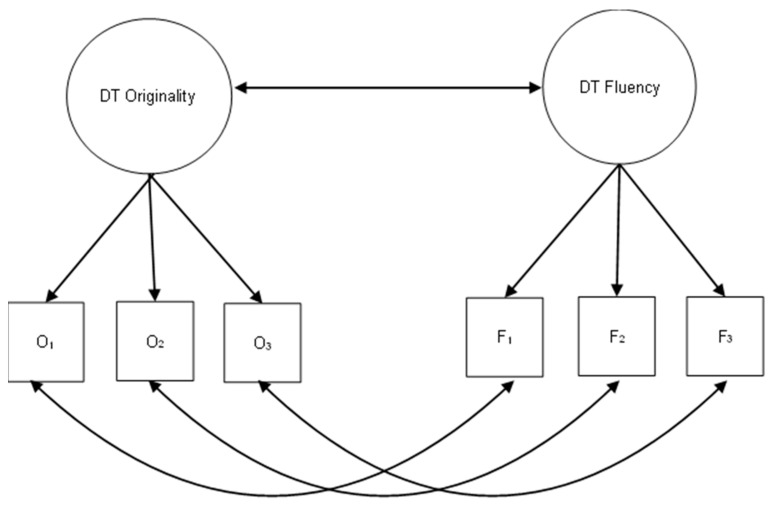
Graphical representation of the Structural Equation Model (SEM) used to assess the correlation between originality and fluency across the different scoring methods.

Next, we fitted latent models using lavaan to examine the latent correlation between originality and fluency (Rosseel 2012). Additionally, we investigated the correlations among all originality factors derived from the different scoring methods. Each model included a latent factor for originality where the originality scores of the three AUTs–varying by scoring method—served as observed indicators, and a latent factor for fluency using the same three fluency scores as observed indicators in all models. While a direct comparison of observed scores for each task would have been possible, we chose instead to reduce the number of comparisons by examining the latent factor of originality—an approach commonly used to aggregate originality scores derived from different items. It is important to note that originality and fluency scores are interdependent, as they are derived from the same task. Thus, to improve the robustness of results, we modeled correlations between originality and fluency at both the item (residual) and the latent level (Saretzki et al. 2024b). A graphical representation of the fitted models is provided in Figure 1. Finally, we examined the latent correlation between originality and fluency at the latent level.

It should be noted that we did not apply IRT to max-scoring because this approach selects responses with the highest average ratings but does not account for variability in individual rater scores. For example, two responses might each have an average rating of 4, yet their rater patterns could differ significantly (e.g., R1 = 3, R2 = 4, R3 = 5 vs. R1 = 5, R2 = 4, R3 = 3). Although both responses yield a mean of 4, choosing one as the “best answer” would potentially distort parameter estimation under an IRT framework. Discrepancies in which raters assign the highest or lowest score can create inconsistencies in how IRT models estimate item difficulty and rater discrimination. Hence, using IRT with max-scoring could undermine the validity of this scoring approach.

Aim 3. We simulated missing data in both the individual response and snapshot scoring datasets. For the individual response dataset, which originally had three raters, we randomly assigned each response to be rated by two of the three raters (corresponding to 33% missing data). For the snapshot dataset, which had five raters, we randomly assigned each participant’s data to be rated by two of the five raters (corresponding to 60% missing data). In both cases, the remaining rater columns were included as missing data, ensuring that each response (for individual response scoring) or participant (for snapshot scoring) ended up with exactly two rater scores. We then re-ran the analyses from Aims 1 and 2 on these reduced datasets to evaluate how introduced sparsity affected reliability. Finally, we examined the correlations between the latent scores obtained from the complete datasets with those from the simulated datasets to assess how robust each scoring approach was to missing data (Primi et al. 2019).

## 3. Results

### 3.1. Aim 1: Applying IRT to Individual Response Scoring and Snapshot Scoring

As a preliminary step, we tested unidimensionality through parallel analysis and by examining the ratio of the first to second eigenvalue. Parallel analysis was conducted to determine the appropriate number of factors. This method compares the eigenvalues from the observed data with those generated from randomly simulated datasets of the same size. Only factors with eigenvalues exceeding the corresponding random eigenvalues are retained. The results indicated that only the first factor’s eigenvalue surpassed the simulated threshold, suggesting a one-factor solution. Moreover, the ratio of the first to second eigenvalue was greater than 4 in both datasets (100,790,113 for individual response scoring, 34.21 for snapshot scoring), supporting the unidimensionality of data. These preliminary analyses provide initial support for the applicability of IRT models to both individual response scoring and snapshot scoring.

Next, we compared the model fit of several IRT models separately for individual response scoring and snapshot scoring (see Table 1). Based on the AIC, the Graded Response Model (GRM) emerged as the best-fitting model for both scoring procedures. Based on the BIC, the pattern was less clear: For individual responses scoring, the Constrained Graded Response Model showed the best fit closely followed by the GRM, whereas for snapshot scoring the Graded Rating Scale Model had the lowest BIC. However, previous studies reported the AIC as the main criterion for model selection (Barbot et al. 2023; Forthmann et al. 2023). Interestingly, the MFRM did not show a better fit for either scoring procedure compared to the models already implemented in the jrt package. Overall, we considered the GRM as the best-fitting model to use in subsequent analyses.

We then examined the rater parameters within the GRM: the intercept (threshold) parameters (d) and the discrimination parameters (a). The parameterization of the GRM implemented in the jrt package is referred to as slope-intercept parameterization (Forthmann et al. 2023). Lower (more negative) intercept values indicate higher rater severity, meaning responses must exhibit a higher level of the latent trait (originality) to receive a particular score. Conversely, higher intercepts reflect greater leniency. We observed substantial variability across the three raters’ severities in individual response scoring. Looking at the intercept *d*_4_ (which represents the threshold from moving for a score of 4 to a score of 5), one rater had a strongly negative intercept (*d*_4_ = −8.14), indicating this rater was the most severe, another had a moderate intercept (*d*_4_ = −5.48), and one had a comparatively higher intercept (*d*_4_ = −3.84), implying greater leniency. In contrast, severity was more homogeneous in snapshot scoring, where the intercepts *d*_4_ ranged from −4.45 to −5.70.

In addition to intercepts, the discrimination parameter *a* indicates how well a rater distinguishes among different levels of latent originality. We found relatively consistent ranges in both scoring methods.

Finally, we checked raters’ infit and outfit statistics. All values fell within the recommended ranges. These results indicate acceptable rater-level fit under both scoring methods. All parameter estimates can be found in Table 2.

**Figure 2:**
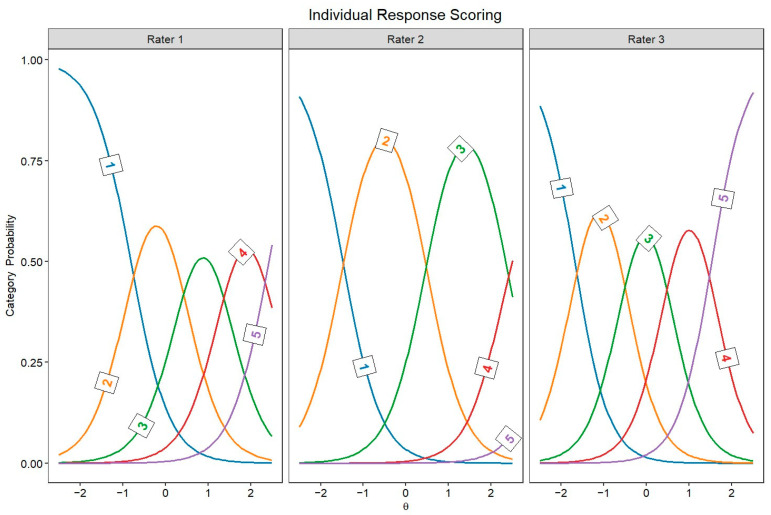
Judge category curves for individual response scoring raters (Θ = latent trait).

**Figure 3:**
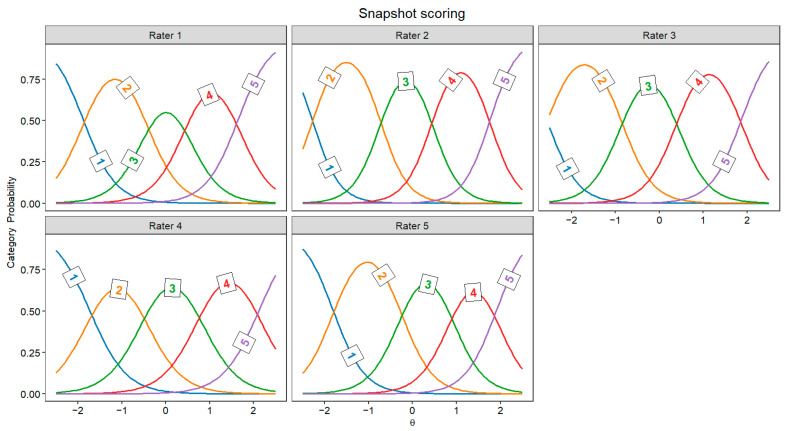
Judge category curves for all snapshot scoring raters (Θ = latent trait).

Figure 2 and Figure 3 display the JCCs, which graphically represent the probability of each score for every rater as a function of latent originality, separately for every rater. In both scoring approaches, these category curves were *sorted*, meaning the thresholds monotonically increased across the five response categories (1 to 5). This pattern indicates that higher categories indeed require higher levels of originality.

**Figure 4:**
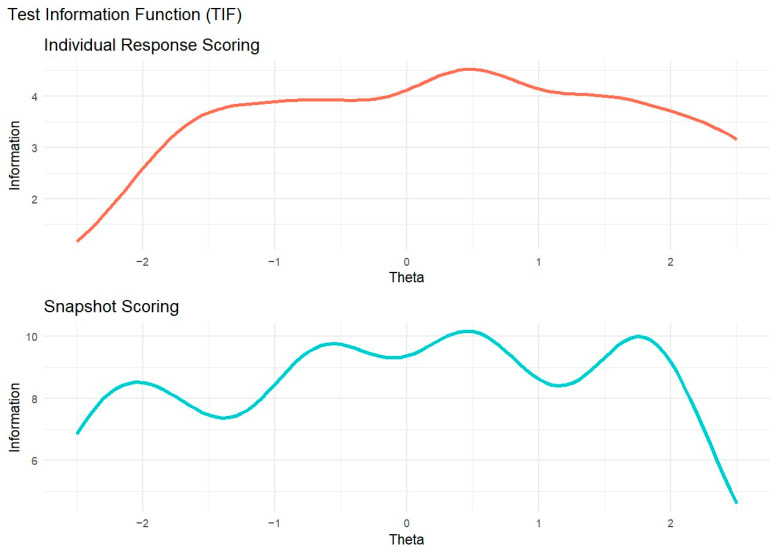
Test information function for individual response scoring and snapshot scoring.

We examined the Total Information Function (TIF) for each model (Figure 4). In individual response scoring, TIF values were relatively stable but peaked near *θ* = 0.5. However, the information sharply declines below *θ* = −1.5. This result suggests that raters provide the most precise information around *θ* = 0.5, while raters are less effective at differentiating responses with lower levels of originality. In snapshot scoring, the TIF showed the opposite pattern: Beyond *θ* = 2, information dropped, suggesting that raters provided limited precision in distinguishing between highly original ideational pools.

### 3.2. Aim 2: Comparing Scoring Approaches

At a descriptive level, we examined the internal consistency of DT scores across all scoring methods using reliability coefficients: Cronbach’s *α* (Cronbach 1951), McDonald’s *ω* (McDonald 1999), and Hancock’s *H* (Hancock and Mueller 2001). These coefficients varied widely depending on the scoring method (see Table 3). Snapshot scoring and max-5 scoring showed the highest reliability, with values above the recommended 0.70 cutoff (DeVellis 1991). In contrast, average scores, max-1 scores, and max-2 scores yielded reliability coefficients below 0.70. Notably, the reliability of the IRT-adjusted scores for both individual response scoring and snapshot scoring was almost identical to that of the corresponding unadjusted scores.

The fit indices for all latent models are reported in Table S1 (Supplementary Materials in OSF). Regarding discriminant validity, the latent correlation between originality and fluency differed considerably among scoring methods (see Table 4). Average scores displayed the lowest correlation (*r* = −0.02, *p* = .85), whereas max-4 scores had the strongest correlation (*r* = 0.57, *p* < .001). For both individual response scoring and snapshot scoring, correlations between fluency and IRT-adjusted vs. unadjusted originality scores were essentially the same. Finally, we observed that for both individual response scoring and snapshot scoring, the IRT-adjusted and non-adjusted scores correlated with *r* > 0.99, indicating they were nearly identical in practice.

### 3.3. Aim 3: Simulating Missing Data

Following the same procedures used for aims 1 and 2, we re-ran all the analyses on the dataset with simulated missing data. In terms of internal consistency, reliability indices were lowered by about 0.05 and thus mostly fell below the recommended cut-off of 0.70 (see Table 3), except for max-5 scoring (*α* = 0.71; *ω* = 0.71; *H* = 0.71). Notably, the difference between IRT-adjusted and non-adjusted scores remained negligible for both average scoring and snapshot scoring. Fit indices for all latent models in the simulated missingness dataset are reported in Table S1 (Supplementary Materials).

Finally, we compared the correlations between the latent originality scores derived from the complete datasets and those obtained from IRT-adjusted and non-adjusted scores in the simulated datasets (see Table 5). For average scoring, the original dataset’s latent originality scores correlated *r* = 0.97 with both the IRT-adjusted and non-adjusted scores under missingness. A similar pattern emerged for snapshot scoring, in which the complete dataset’s originality factor correlated *r* = 0.95 with the adjusted and *r* = 0.94 with the unadjusted scores in the simulated dataset.

## 4. Discussion

Previous work on rater-based DT scoring has focused on understanding the relevance of rater characteristics (Benedek et al. 2016; Ceh et al. 2022), providing guidelines for improving rater agreement (Silvia et al. 2008), and reducing the rating burden (Forthmann et al. 2017a). More recently, the IRT framework has been proposed as a possible way to examine differences in individual judgments of DT tasks, allowing the estimation of more reliable originality scores by adjusting for potential differences in raters’ severity and discrimination (Myszkowski and Storme 2019; Primi et al. 2019). In the present study, we aim to expand research on the application of IRT models to DT scoring using a pre-existent dataset (Forthmann et al. 2017b).

Our first aim was to determine the best IRT model for both individual response scoring and snapshot scoring. While IRT models have already been successfully applied to individual response scoring (Barbot et al. 2023; Forthmann et al. 2023; Primi et al. 2019), no previous study has yet applied these models to snapshot scoring. From a statistical perspective, both scoring approaches are similar in that raters score the tasks on a Likert scale, so we expected that IRT models could be applied to both scoring approaches. The jrt package (Myszkowski 2021) facilitates direct comparisons among the most common polytomous IRT models. However, at the moment, the jrt package does not allow for the incorporation of multiple facets (e.g., both items and raters). For this reason, we compared JRT models with the MFRM (Primi et al. 2019). Using the AIC as our primary benchmark to evaluate model fit (Barbot et al. 2023; Forthmann et al. 2023), we found that the GRM provided the best fit for both individual response scoring and snapshot scoring. Interestingly, the MFRM—which allows the estimation of task parameters as well as rater parameters—did not show a better fit. A likely explanation is that our dataset included only three tasks, whereas MFRM may perform better with a greater number of tasks. For instance, Primi et al. (2019) included six objects in their AUT, which may have introduced greater variability in task ratings, allowing the MFRM to capture and handle this variability.

The GRM allowed us to compare raters’ behavior in individual response scoring and snapshot scoring. In particular, the TIF highlighted notable differences between these two approaches. In individual response scoring, raters provided more homogeneous information; however, this information steeply declined for lower-quality responses. This pattern likely reflects that some raters were more severe in assigning high scores, whereas lower scores were more frequently given. In other words, raters may have focused on distinguishing highly original responses while being less attentive to differences among low-originality responses. Conversely, in snapshot scoring, the TIF revealed the opposite trend: information varied more across different levels of originality but declined sharply for highly creative responses. This suggests that raters in snapshot scoring may have been more lenient in assigning higher scores, possibly because considering several ideas together may be more likely to give the impression of a highly creative performance than a single response. Therefore, snapshot ratings might benefit from a more differentiated response scale (e.g., 7 points instead of 5 points) to better discriminate between the most creative performances. It should be noted that, in individual response scoring, the TIF could be highly influenced by a rater (i.e., rater 2 in Figure 2) who was more severe than the others, attributing only a few high scores. Moreover, the TIF is also influenced by the number of raters involved, thus the disparity in the number of raters across individual response scoring and snapshot scoring may prevent a direct comparison of the two approaches.

Moving to the second aim of our study, we assessed the reliability of AUT scores derived from different scoring approaches including IRT-adjusted and unadjusted versions of average scoring (for both individual response scoring and snapshot scoring), as well as max-scoring. All these methods are widely used in creativity research (Reiter-Palmon et al. 2019; Saretzki et al. 2024a). However, systematic comparisons of different scoring approaches have only recently gained attention (Saretzki et al. 2024b). Similarly to previous findings (Primi et al. 2019), our results showed that IRT-adjusted scores were practically identical to unadjusted scores, with a correlation of *r* > 0.99 for both individual response scoring and snapshot scoring. This finding suggests that, although raters may differ in terms of severity and discrimination, the relative ranking of individual responses and ideational pools remains stable across raters and therefore does not affect correlational relationships. It is possible that the variance in rater severity and discrimination was not large enough to alter response rankings once scores were aggregated, which might indicate that aggregation methods (i.e., average, snapshot, or max-scoring) are already compensating for rater differences. This result is also reflected in the high inter-rater reliability in both individual response scoring and snapshot scoring, as shown by the ICC. Ultimately, our findings indicate that, in this dataset, rater differences in severity and discrimination do not appear to affect responses’ ranking. However, adjusted scores may still play a greater role in contexts where absolute rater agreement is required, such as when focusing on the absolute level of creativity scores rather than their relative rankings.

In terms of internal consistency, snapshot scoring had the highest reliability coefficients. However, it should be noted that in snapshot scoring five raters were involved, while in individual response scoring only three raters evaluated the tasks. Max-scoring from three to five responses also showed adequate internal consistency, slightly higher than average scoring. Max-1 scores showed poor internal reliability, a result that is consistent with previous findings (Saretzki et al. 2024b).

We further examined the confounding effect of response fluency (Forthmann et al. 2021; Saretzki et al. 2024b) across the different scoring methods. We observed that average scores were not correlated to fluency. For max-n scoring, the more responses were considered, the higher the correlation with fluency. This finding is interesting as one might think that there might be a trade-off between a focus on generating many ideas and producing a few highly creative ideas. Still, this result is consistent with previous studies (Saretzki et al. 2024b) and suggests that having many ideas is an important precondition for generating several highly creative ideas, as presumed by the equal odds model (Forthmann 2024). The correlation between snapshot scores and fluency was small, in line with previous research (Saretzki et al. 2024b; Silvia et al. 2008). As for max-scoring, snapshot scoring may also reflect the intrinsic association between fluency and originality (Forthmann 2024). Moreover, this result further suggests that raters are not overly influenced by the number of responses provided by participants when providing a snapshot score of the entire ideational pool (i.e., they do not consistently attribute higher originality scores to participants who provided a high number of responses).

Finally, the third aim of our study was to examine the utility of IRT models in the case of missing data. To reduce the number of resources and time required to score DT tasks, IRT models have been considered a potential solution to manage the situation where raters only evaluate a few of the responses that participants provided (Forthmann et al. 2023; Myszkowski and Storme 2019). Our simulated missingness scenario mainly confirmed our previous results: Not only do IRT-adjusted scores appear to have similar internal consistency to unadjusted scores, but even when comparing these scores with the original ones from the complete dataset, the results are practically identical. While Primi et al. (2019) found that the MFRM slightly improved the correlations between the original scores and the scores obtained from the missingness dataset, we did not find similar beneficial effects of the GRM. Moreover, the decrease in reliability was similar for adjusted and unadjusted scores. It is interesting to note that max-5 scoring was the only scoring approach that retained an adequate level of internal consistency in the simulated missing dataset, even surpassing snapshot scoring.

Taken together, our results confirm that IRT models can be effectively applied to DT task scores, not only for individual response scoring but also for snapshot scoring. Moreover, these models provide a valuable tool for examining rater behavior and identifying potential differences in discrimination and severity (Myszkowski and Storme 2019). Interestingly, raters exhibited opposite scoring patterns in the two approaches: They were more stringent when assigning high scores in individual response scoring but more lenient in snapshot scoring. IRT-adjusted scores remained psychometrically similar to unadjusted scores, at least in terms of internal consistency and correlation with fluency, suggesting that under conditions of high inter-rater agreement, differences in rater severity and leniency may have had only a limited impact on the scores. This pattern persisted even when missing ratings were introduced into the dataset. It is important to note that in our dataset, inter-rater reliability, as measured by the ICC, was high. In this context, differences in rater severity and scoring consistency may not significantly impact on the overall ranking of responses, which could explain the similar reliability indices and the strong correlation between adjusted and unadjusted scores.

### Limitations and Future Directions

A major limitation of this study is the lack of measures to assess concurrent validity (e.g., creative behavior, creative self-beliefs, and openness to experience), which is an essential component when comparing different scoring methods (Saretzki et al. 2024b). Moreover, the present study only focused on the application of IRT to DT originality. However, also DT fluency and flexibility scores could be examined within the IRT framework by using specific models that account for count data (Myszkowski 2024).

A second limitation is that individual response scoring and snapshot scoring involve a different number of raters. While our simulated missingness dataset ensured an equal number of raters in both approaches, future research should consider the potential impact of the number of raters on the reliability of DT scores. Moreover, further experimental studies are needed to replicate the findings of our simulation and verify that planned missing data designs yield reliable results for DT originality assessment. Such studies may also examine whether this design offers additional benefits, such as reducing the potential fatigue associated with scoring multiple tasks (Forthmann et al. 2017a).

This study represents the first systematic comparison of IRT models applied to different DT scoring methods (i.e., individual response scoring and snapshot scoring). Our findings suggest that IRT-adjusted and non-adjusted scores overlap substantially, at least when inter-rater agreement is high. However, further research is needed to determine under which conditions differences in rater severity and discrimination undermine the psychometric quality of DT scores. For instance, IRT models could be extended beyond the AUT to other DT tasks (e.g., figural tasks). Rater behavior may vary depending on the task type, and IRT models could help identify and account for such differences. Such studies could help identify when the use of IRT is essential to minimize bias due to rater variability, thereby guiding researchers in deciding when IRT models are especially preferable over more traditional approaches.

Additionally, given the substantial time invested in training raters and providing scoring guidelines, future studies could explore differences between novice and expert raters. It is reasonable to expect greater variability in severity and discrimination among novice raters. IRT models might handle this variability, potentially making novice raters as reliable as expert raters.

Finally, in this study, we only explored the behavior of human raters. However, given the recent development of different tools for the automated scoring of creativity tasks through Large Language Models (e.g., Zielińska et al. 2023), future research could extend the application of IRT models to automated scores. IRT could represent a sophisticated framework for examining differences and similarities between human and non-human raters.

## 5. Conclusions

IRT models offer a promising new approach for the statistical analysis of DT data. While the present study found that IRT-adjusted scores exhibited psychometric properties similar to unadjusted scores, it also demonstrated the practical advantages of using IRT to examine rater differences in both individual response scoring and snapshot scoring. IRT models allow for precise quantification of differences in rater severity and discrimination and enhance the interpretability of scoring processes. The development of computational tools, such as the jrt package (Myszkowski 2021), will further facilitate the application of IRT in DT research, supporting the integration of this approach in creativity research.

## Figures and Tables

**Table 1:** Comparison of all the Item Response Theory (IRT) models tested for individual response scoring and snapshot scoring.

	Individual Response Scoring		Snapshot Scoring	
Model	AIC	BIC	AIC	BIC
Rating Scale Model	34,925.89	34,971.16	6669.37	6708.98
Generalized Rating Scale Model	34,299.33	34,363.88	6635.26	6696.89
Partial Credit Model	33,721.05	33,805.11	6619.11	6711.55
Generalized Partial Credit Model	33,710.67	33,807.66	6607.11	6717.16
Constrained Graded Rating Scale Model	33,705.17	35,006.43	6657.57	6697.18
Graded Rating Scale Model	34,262.57	34,320.77	6625.81	**6683.03**
Constrained Graded Response Model	33,705.17	**33,789.23**	6603.03	6695.47
Graded Response Model	**33,703.75**	33,800.75	**6602.47**	6712.52
Many Facet Rasch Model	34,863.84	34,928.51	7137.61	7177.25

Note. AIC = Akaike Information Criterion. BIC = Bayesian Information Criterion. The lowest AIC and BIC for each scoring procedure are in bold.

**Table 2:** Intercept parameters (*d*), discrimination parameters (*a*), infit, and outfit for all raters.

		*a*	*d*1	*d*2	*d*3	*d*4	Outfit	Infit
Individual Response Scoring	Rater 1	2.26	1.83	−0.87	−3.12	−5.48	0.68	0.70
	Rater 2	2.26	3.43	−1.07	−5.33	−8.14	0.77	0.78
	Rater 3	2.51	4.24	1.34	−1.21	−3.84	0.64	0.65
Snapshot Scoring	Rater 1	2.71	5.09	1.20	−1.26	−4.45	0.81	0.82
	Rater 2	3.24	7.39	2.36	−1.43	−5.70	0.75	0.77
	Rater 3	2.84	7.27	2.44	−1.14	−5.29	0.80	0.80
	Rater 4	2.41	4.17	1.18	−1.86	−5.10	0.84	0.85
	Rater 5	2.80	5.05	0.72	−2.47	−5.33	0.79	0.80

**Table 3:** Reliability indices (Cronbach’s *α*, McDonald’s *ω*, and Hancock’s *H*) in the complete and simulated missingness dataset.

	α	ω	H
**Fluency**	0.85 [0.80; 0.88]	0.85 [0.80; 0.89]	0.87 [0.83; 0.92]
Complete dataset			
Average scoring	0.67 [0.57; 0.75]	0.67 [0.57; 0.75]	0.69 [0.60; 0.78]
IRT-adjusted average scoring	0.68 [0.57; 0.76]	0.68 [0.58; 0.76]	0.68 [0.60; 0.78]
Max-1 scoring	0.56 [0.43; 0.66]	0.56 [0.43; 0.65]	0.56 [0.46; 0.74]
Max-2 scoring	0.65 [0.55; 0.73]	0.65 [0.56; 0.73]	0.66 [0.59; 0.78]
Max-3 scoring	0.70 [0.62; 0.77]	0.71 [0.63; 0.77]	0.72 [0.65; 0.84]
Max-4 scoring	0.72 [0.64; 0.79]	0.72 [0.64; 0.79]	0.73 [0.66; 0.82]
Max-5 scoring	0.73 [0.64; 0.79]	0.73 [0.64; 0.78]	0.73 [0.67; 0.81]
Snapshot scoring	0.74 [0.66; 0.80]	0.74 [0.66; 0.80]	0.74 [0.67; 0.83]
IRT-adjusted snapshot scoring	0.74 [0.66; 0.80]	0.74 [0.64; 0.80]	0.74 [0.67; 0.83]
Simulated missingness dataset			
Average scoring	0.64 [0.52; 0.73]	0.64 [0.53; 0.73]	0.65 [0.56; 0.79]
IRT-adjusted average scoring	0.64 [0.52; 0.72]	0.65 [0.54; 0.73]	0.65 [0.56; 0.77]
Max-1 scoring	0.50 [0.36; 0.61]	0.50 [0.37; 0.61]	0.52 [0.42; 0.95]
Max-2 scoring	0.59 [0.47; 0.67]	0.60 [0.49; 0.68]	0.61 [0.54; 0.80]
Max-3 scoring	0.67 [0.58; 0.74]	0.67 [0.57; 0.74]	0.68 [0.60; 0.80]
Max-4 scoring	0.68 [0.58; 0.75]	0.68 [0.59; 0.75]	0.68 [0.60; 0.77]
Max-5 scoring	0.71 [0.62; 0.78]	0.71 [0.62; 0.77]	0.71 [0.64; 0.80]
Snapshot scoring	0.69 [0.59; 0.76]	0.69 [0.59; 0.76]	0.69 [0.60; 0.78]
IRT-adjusted snapshot scoring	0.69 [0.60; 0.76]	0.69 [0.59; 0.76]	0.70 [0.62; 0.79]

Note. The 95% Confidence Intervals (CIs) based on 1000 bootstrap samples are reported in brackets.

**Table 4:** Correlations between all latent factors of originality and fluency.

	1.	2.	3.	4.	5.	6.	7.	8.	9.
1. Average scoring	-								
2. IRT-adjusted average scoring	>0.99 **	-							
3. Max-1 scoring	0.75 **	0.74 **	-						
4. Max-2 scoring	0.76 **	0.75 **	0.96 **	-					
5. Max-3 scoring	0.74 **	0.74 **	0.93 **	0.99 **	-				
6. Max-4 scoring	0.74 **	0.73 **	0.92 **	0.97 **	0.99 **	-			
7. Max-5 scoring	0.76 **	0.76 **	0.92 **	0.96 **	0.98 **	0.99 **	-		
8. Snapshot scoring	0.79 **	0.79 **	0.85 **	0.86 **	0.85 **	0.84 **	0.85 **	-	
9. IRT-adjusted snapshot scoring	0.80 **	0.80 **	0.84 **	0.86 **	0.84 **	0.83 **	0.84 **	>0.99 **	-
10. Fluency	−0.02	−0.02	0.40 **	0.48 **	0.54 **	0.57 **	0.55 **	0.29 **	0.28 *

Note. It should be noted that correlations between originality and fluency were estimated separately in each latent model. * *p* < .01; ** *p* < .001.

**Table 5:** Correlations between the scores between the complete dataset and the simulated missing dataset (average scoring and snapshot scoring).

		Simulated Dataset			
		Average Scoring	IRT-Adjusted Average Scoring	Snapshot Scoring	IRT-Adjusted Snapshot Scoring
Complete dataset	Average scoring	0.97	0.97	0.73	0.75
	IRT-adjusted average scoring	0.97	0.98	0.72	0.74
	Snapshot scoring	0.75	0.76	0.94	0.95
	IRT-adjusted snapshot scoring	0.75	0.76	0.94	0.94

Note. All correlations are significant with *p* < .001.

## Data Availability

The data are available at https://osf.io/a9qnc/ (accessed on 27 September 2024).

## Supplementary Materials

The following supporting information can be downloaded at: https://www.mdpi.com/article/10.3390/jintelligence13060069/s1, Table S1: Fit indices for all latent models in the complete and simulated dataset.

## Abbreviations

The following abbreviations are used in this manuscript:

DT	Divergent Thinking
IRT	Item Response Theory
AUT	Alternate Uses Task
JRT	Judge Response Theory
ICC	Intraclass Correlation Coefficient
MFRM	Many-Facet Rasch Model
AIC	Akaike Information Criterion
BIC	Bayesian Information Criterion
JCC	Judge Category Curves
TIF	Test Information Function
EAP	Expected A Posteriori
GRM	Graded Response Model

## References

[B1-jintelligence-13-00069] Amabile Teresa M. (1982). Social psychology of creativity: A consensual assessment technique. Journal of Personality and Social Psychology.

[B2-jintelligence-13-00069] Andrich David (1978). A Rating Formulation for Ordered Response Categories. Psychometrika.

[B3-jintelligence-13-00069] Baer John, Kaufman James C., Gentile Claudia A. (2004). Extension of the Consensual Assessment Technique to Nonparallel Creative Products. Creativity Research Journal.

[B4-jintelligence-13-00069] Barbot Baptiste, Kaufman James C., Myszkowski Nils (2023). Creativity with 6 Degrees of Freedom: Feasibility Study of Visual Creativity Assessment in Virtual Reality. Creativity Research Journal.

[B5-jintelligence-13-00069] Benedek Mathias (2024). On the relationship between creative potential and creative achievement: Challenges and future directions. Learning and Individual Differences.

[B6-jintelligence-13-00069] Benedek Mathias, Mühlmann Caterina, Jauk Emanuel, Neubauer Aljoscha C. (2013). Assessment of divergent thinking by means of the subjective top-scoring method: Effects of the number of top-ideas and time-on-task on reliability and validity. Psychology of Aesthetics, Creativity, and the Arts.

[B7-jintelligence-13-00069] Benedek Mathias, Nordtvedt Nora, Jauk Emanuel, Koschmieder Corinna, Pretsch Jürgen, Krammer Georg, Neubauer Aljoscha C. (2016). Assessment of creativity evaluation skills: A psychometric investigation in prospective teachers. Thinking Skills and Creativity.

[B8-jintelligence-13-00069] Botella Marion, Zenasni Franck, Lubart Todd (2018). What Are the Stages of the Creative Process? What Visual Art Students Are Saying. Frontiers in Psychology.

[B9-jintelligence-13-00069] Ceh Simon Majed, Edelmann Carina, Hofer Gabriela, Benedek Mathias (2022). Assessing Raters: What Factors Predict Discernment in Novice Creativity Raters?. The Journal of Creative Behavior.

[B10-jintelligence-13-00069] Clark Philip M., Mirels Herbert L. (1970). Fluency as a pervasive element in the measurement of creativity. Journal of Educational Measurement.

[B11-jintelligence-13-00069] Cronbach Lee J. (1951). Coefficient alpha and the internal structure of tests. Psychometrika.

[B12-jintelligence-13-00069] DeVellis Robert F. (1991). Scale Development.

[B13-jintelligence-13-00069] Eckes Thomas (2011). Introduction to Many-Facet Rasch Measurement.

[B14-jintelligence-13-00069] Forthmann Boris (2024). Disentangling Quantity and Quality in the Assessment of Creative Productions. Creativity Research Journal.

[B15-jintelligence-13-00069] Forthmann Boris, Doebler Philipp (2022). Fifty years later and still working: Rediscovering Paulus et al.’s (1970) automated scoring of divergent thinking tests. Psychology of Aesthetics, Creativity, and the Arts.

[B16-jintelligence-13-00069] Forthmann Boris, Goecke Benjamin, Beaty Roger E. (2023). Planning Missing Data Designs for Human Ratings in Creativity Research: A Practical Guide. Creativity Research Journal.

[B17-jintelligence-13-00069] Forthmann Boris, Szardenings Carsten, Dumas Denis (2021). On the Conceptual Overlap between the Fluency Contamination Effect in Divergent Thinking Scores and the Chance View on Scientific Creativity. The Journal of Creative Behavior.

[B18-jintelligence-13-00069] Forthmann Boris, Holling Heinz, Zandi Nima, Gerwig Anne, Çelik Pınar, Storme Martin, Lubart Todd (2017a). Missing creativity: The effect of cognitive workload on rater (dis-)agreement in subjective divergent-thinking scores. Thinking Skills and Creativity.

[B19-jintelligence-13-00069] Forthmann Boris, Holling Heinz, Çelik Pınar, Storme Martin, Lubart Todd (2017b). Typing Speed as a Confounding Variable and the Measurement of Quality in Divergent Thinking. Creativity Research Journal.

[B20-jintelligence-13-00069] Forthmann Boris, Paek Sue Hyeon, Dumas Denis, Barbot Baptiste, Holling Heinz (2020). Scrutinizing the basis of originality in divergent thinking tests: On the measurement precision of response propensity estimates. British Journal of Educational Psychology.

[B21-jintelligence-13-00069] Guilford Joy Paul (1950). Creativity. American Psychologist.

[B22-jintelligence-13-00069] Guilford Joy Paul (1967). The Nature of Human Intelligence.

[B23-jintelligence-13-00069] Hancock Gregory, Mueller Ralph O., Cudeck Robert, Toit Stephen du, Sörbom Dag (2001). Rethinking construct reliability within latent variable systems. Structural Equation Modeling: Present and Future—A Festschrift in Honor of Karl Jöreskog.

[B24-jintelligence-13-00069] Koo Terry K., Li Mae Y. (2016). A Guideline of Selecting and Reporting Intraclass Correlation Coefficients for Reliability Research. Journal of Chiropractic Medicine.

[B25-jintelligence-13-00069] Lubart Todd I. (2001). Models of the Creative Process: Past, Present and Future. Creativity Research Journal.

[B26-jintelligence-13-00069] Masters Geoff N. (1982). A Rasch Model for Partial Credit Scoring. Psychometrika.

[B27-jintelligence-13-00069] McDonald Roderick P. (1999). Test Theory: A Unified Treatment.

[B28-jintelligence-13-00069] Muraki Eiji (1993). Information Functions of the Generalized Partial Credit Model. Applied Psychological Measurement.

[B29-jintelligence-13-00069] Myszkowski Nils (2021). Development of the R library “jrt”: Automated item response theory procedures for judgment data and their application with the consensual assessment technique. Psychology of Aesthetics, Creativity, and the Arts.

[B30-jintelligence-13-00069] Myszkowski Nils (2024). Item Response Theory for Creativity Measurement.

[B31-jintelligence-13-00069] Myszkowski Nils, Storme Martin (2019). Judge response theory? A call to upgrade our psychometrical account of creativity judgments. Psychology of Aesthetics, Creativity, and the Arts.

[B32-jintelligence-13-00069] Patterson John D., Merseal Hannah M., Johnson Dan R., Agnoli Sergio, Baas Matthijs, Baker Brendan S., Barbot Baptiste, Benedek Mathias, Borhani Khatereh, Chen Qunlin (2023). Multilingual semantic distance: Automatic verbal creativity assessment in many languages. Psychology of Aesthetics, Creativity, and the Arts.

[B33-jintelligence-13-00069] Plucker Jonathan A., Qian Meihua, Wang Shujuan (2011). Is Originality in the Eye of the Beholder? Comparison of Scoring Techniques in the Assessment of Divergent Thinking. The Journal of Creative Behavior.

[B34-jintelligence-13-00069] Primi Ricardo, Silvia Paul J., Jauk Emanuel, Benedek Mathias (2019). Applying many-facet Rasch modeling in the assessment of creativity. Psychology of Aesthetics, Creativity, and the Arts.

[B35-jintelligence-13-00069] R Core Team (2024). R: A Language and Environment for Statistical Computing.

[B36-jintelligence-13-00069] Reiter-Palmon Roni, Forthmann Boris, Barbot Baptiste (2019). Scoring divergent thinking tests: A review and systematic framework. Psychology of Aesthetics, Creativity, and the Arts.

[B37-jintelligence-13-00069] Robitzsch Alexander, Kiefer Thomas, Wu Margaret (2024). TAM: Test Analysis Modules. R Package Version 4.2-21. https://CRAN.R-project.org/package=TAM.

[B38-jintelligence-13-00069] Rosseel Yves (2012). lavaan: An R Package for Structural Equation Modeling. Journal of Statistical Software.

[B39-jintelligence-13-00069] Said-Metwaly Sameh, Taylor Christa L., Camarda Anaëlle, Barbot Baptiste (2022). Divergent thinking and creative achievement—How strong is the link? An updated meta-analysis. Psychology of Aesthetics, Creativity, and the Arts.

[B40-jintelligence-13-00069] Samejima Fumi (1968). Estimation of latent ability using a response pattern of graded scores. ETS Research Bulletin Series.

[B41-jintelligence-13-00069] Saretzki Janika, Forthmann Boris, Benedek Mathias (2024a). A systematic quantitative review of divergent thinking assessments. Psychology of Aesthetics, Creativity, and the Arts.

[B42-jintelligence-13-00069] Saretzki Janika, Andrae Rosalie, Forthmann Boris, Benedek Mathias (2024b). Investigation of response aggregation methods in divergent thinking assessments. The Journal of Creative Behavior.

[B43-jintelligence-13-00069] Silvia Paul J. (2008). Discernment and creativity: How well can people identify their most creative ideas?. Psychology of Aesthetics, Creativity, and the Arts.

[B44-jintelligence-13-00069] Silvia Paul J. (2011). Subjective scoring of divergent thinking: Examining the reliability of unusual uses, instances, and consequences tasks. Thinking Skills and Creativity.

[B45-jintelligence-13-00069] Silvia Paul J., Winterstein Beate P., Willse John T., Barona Christopher M., Cram Joshua T., Hess Karl I., Martinez Jenna L., Richard Crystal A. (2008). Assessing creativity with divergent thinking tasks: Exploring the reliability and validity of new subjective scoring methods. Psychology of Aesthetics, Creativity, and the Arts.

[B46-jintelligence-13-00069] Silvia Paul J., Martin Christopher, Nusbaum Emily C. (2009). A snapshot of creativity: Evaluating a quick and simple method for assessing divergent thinking. Thinking Skills and Creativity.

[B47-jintelligence-13-00069] Smeekens Bridget A., Kane Michael J. (2016). Working memory capacity, mind wandering, and creative cognition: An individual-differences investigation into the benefits of controlled versus spontaneous thought. Psychology of Aesthetics, Creativity, and the Arts.

[B48-jintelligence-13-00069] Wright Ben D., Linacre John Michael (1994). Reasonable mean-square fit values. Rasch Measurement Transactions.

[B49-jintelligence-13-00069] Yu Yuhua, Beaty Roger E., Forthmann Boris, Beeman Mark, Cruz John Henry, Johnson Dan (2023). A MAD method to assess idea novelty: Improving validity of automatic scoring using maximum associative distance (MAD). Psychology of Aesthetics, Creativity, and the Arts.

[B50-jintelligence-13-00069] Zielińska Aleksandra, Lebuda Izabela, Karwowski Maciej (2022). Scaling the Creative Self: An Item Response Theory Analysis of the Short Scale of Creative Self. Creativity Research Journal.

[B51-jintelligence-13-00069] Zielińska Aleksandra, Organisciak Peter, Dumas Denis, Karwowski Maciej (2023). Lost in translation? Not for Large Language Models: Automated divergent thinking scoring performance translates to non-English contexts. Thinking Skills and Creativity.

