# Supplementary material for: Controlling Rater Effects in Divergent Thinking Assessment: An Item Response Theory Approach to Individual Response and Snapshot Scoring
Source: J Intell. 2025 Jun 17;13(6):69. doi: 10.3390/jintelligence13060069 (PMC12194098; doi:10.3390/jintelligence13060069)
Supplement: Supplementary file 1 [file jintelligence-13-00069-s001.zip › jintelligence-3575537-supplementary.pdf]

Supplementary Materials

Table S1

Fit indices for all latent models in the complete and simulated dataset.

Model	χ2	df	p	RMSEA	SRMR	CFI	TLI
Complete dataset							
Average scoring	16.50	5	< .01	.11	.05	.97	.91
IRT-adjusted average scoring	17.11	5	< .01	.11	.05	.97	.90
Max-1-scoring	13.68	5	< .05	.09	.05	.98	.93
Max-2-scoring	14.58	5	< .05	.10	.05	.98	.93
Max-3-scoring	14.09	5	< .05	.10	.05	.98	.95
Max-4-scoring	16.93	5	< .01	.11	.05	.98	.93
Max-5-scoring	18.03	5	< .01	.11	.05	.98	.93
Snapshot scoring	10.21	5	.07	.07	.04	.99	.96
IRT-adjusted snapshot scoring	11.12	5	< .05	.08	.04	.99	.96
Simulated missing data							
Average scoring	13.41	5	< .05	.09	.05	.98	.93
IRT-adjusted average scoring	14.44	5	< .05	.10	.05	.97	.92
Max-1-scoring	8.54	5	.13	.06	.03	.99	.97
Max-2-scoring	10.94	5	.05	.08	.04	.99	.96
Max-3-scoring	13.40	5	< .05	.09	.04	.98	.95
Max-4-scoring	14.57	5	< .05	.10	.05	.98	.94
Max-5-scoring	16.79	5	< .01	.11	.05	.98	.93
Snapshot scoring	6.20	5	.29	.03	.03	1.00	.99
IRT-adjusted snapshot scoring	5.19	5	.39	.01	.03	1.00	1.00

Notes. IRT = Item Response Theory. RMSEA = Root Mean Square Error of Approximation. SRMR = Standardized Root Mean Square Residual. CFI = Comparative Fit Index. TLI = Tucker Lewis Index.
